# Concurrent pulmonary arteriovenous malformation and pulmonary embolism causing stroke: a therapeutic dilemma

**DOI:** 10.1186/s42155-021-00282-y

**Published:** 2022-01-06

**Authors:** Jung Guen Cha, Jihoon Hong

**Affiliations:** Department of Radiology, School of Medicine, Kyungpook National University, 680 Gukchaebosang-ro, Jung-gu, Daegu, 41944 South Korea

**Keywords:** Pulmonary arteriovenous malformation, Pulmonary embolism, Stroke, Antiphospholipid syndrome

## Abstract

**Background:**

As pulmonary arteriovenous malformation (PAVM) include a right-to-left shunt, it can be accompanied by fatal complications such as stroke and brain abscess due to paradoxical embolism. A concurrent PAVM and pulmonary embolism (PE) is a rare condition. Therefore, the sequence of management has not been established.

**Case presentation:**

A 62-year-old female patient was transferred to our hospital with a sporadic simple PAVM and concurrent bilateral PE. On chest computed tomography (CT), the acute PE was extended to the segmental pulmonary artery where the feeding artery of PAVM originated. Despite the anticoagulation, the patient complained of left sided weakness on the fifth day of admission, and magnetic resonance imaging revealed an acute infarction in the right lateral thalamus, which was thought to be caused by paradoxical embolism. This situation could lead to a dilemma between the risk of thrombus migration during PAVM embolization and another embolic event due to delayed shunt occlusion during anticoagulation. After a multidisciplinary discussion, a delayed endovascular embolization was performed for PAVM after confirming the complete resolution of PE with 4 months of anticoagulation. The cause of PE in this patient was eventually diagnosed as antiphospholipid syndrome.

**Conclusion:**

The authors reported a rare case of concurrent PAVM and PE that led to an embolic stroke during hospitalization. This patient was managed with delayed endovascular embolization for PAVM after an anticoagulation for PE and stroke. It is thought to be valuable in deciding for a treatment plan for this rare condition.

## Background

Pulmonary arteriovenous malformation (PAVM), which is associated with hereditary hemorrhagic telangiectasia (HHT) or occurs sporadically, can cause neurologic complications such as stroke and brain abscess through a right-to-left shunt. In the case of ischemic stroke, a common assumption is that it occurs as a result of paradoxical embolism of venous thromboemboli from the systemic venous circulation or PAVM sac (Shovlin et al., 2014). These paradoxical embolisms are more common in patients with higher grade shunts on transthoracic contrast echocardiography (TTCE), which are more likely to be associated with PAVM seen on computed tomography (CT) (Velthuis et al., 2014; Velthuis et al., 2013). Therefore, any PAVM with a feeding artery of ≥2 mm, that is discernible on pulmonary angiography and accessible with catheter technique, is recommended for endovascular embolization (Muller-Hulsbeck et al., 2020). A concurrent PAVM and pulmonary embolism (PE) is a rare condition and the increased concern about paradoxical embolism makes the situation more complex in terms of its treatment. Although there are no disagreements about anticoagulation with endovascular embolization of PAVM as a first-line management for this situation, the timing of embolization has not been clearly established.

## Case presentation

**Fig. 1:**
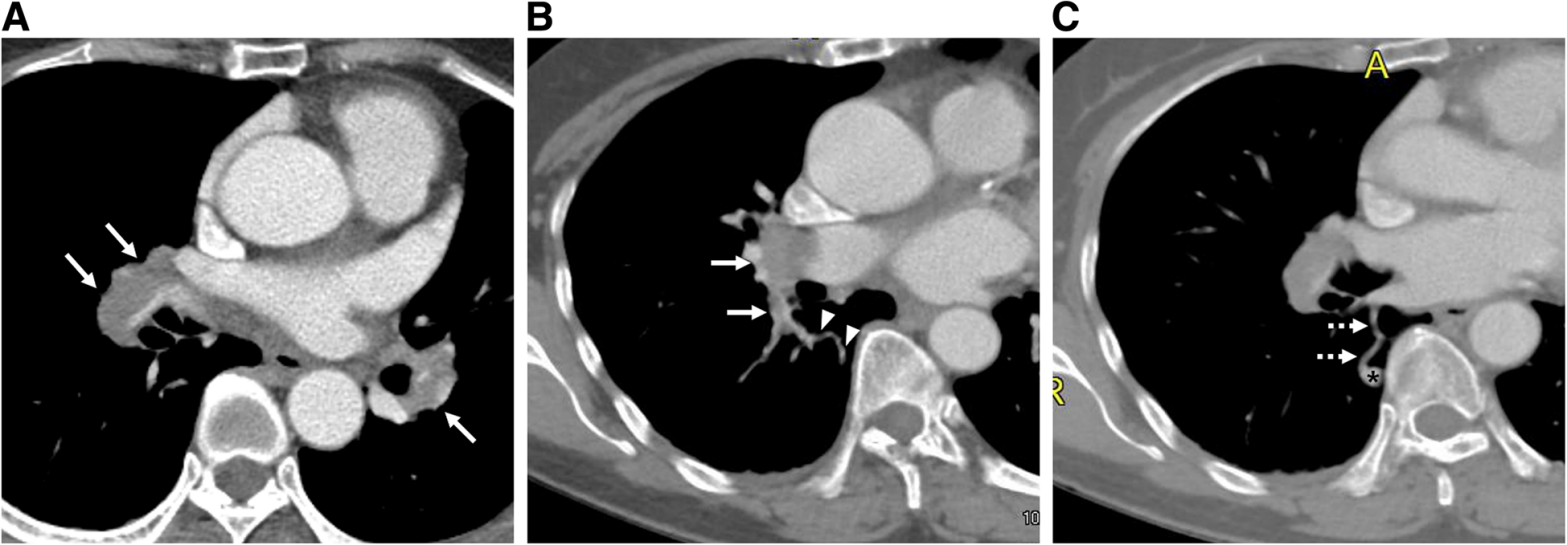
**A** Initially performed computed tomography (CT) image shows acute multifocal pulmonary embolism (PE) involving the bilateral lobar and segmental branches of the pulmonary artery (arrows). **B, C** Multiplanar reconstructed CT images show the angioarchitecture of the pulmonary arteriovenous malformation (PAVM) consisting of the feeding artery (arrowheads), venous sac (asterisk), and draining vein (dashed arrows). Thromboembolisms that spread to the segmental artery from which the feeder originate are also observed (arrows)

**Fig. 2:**
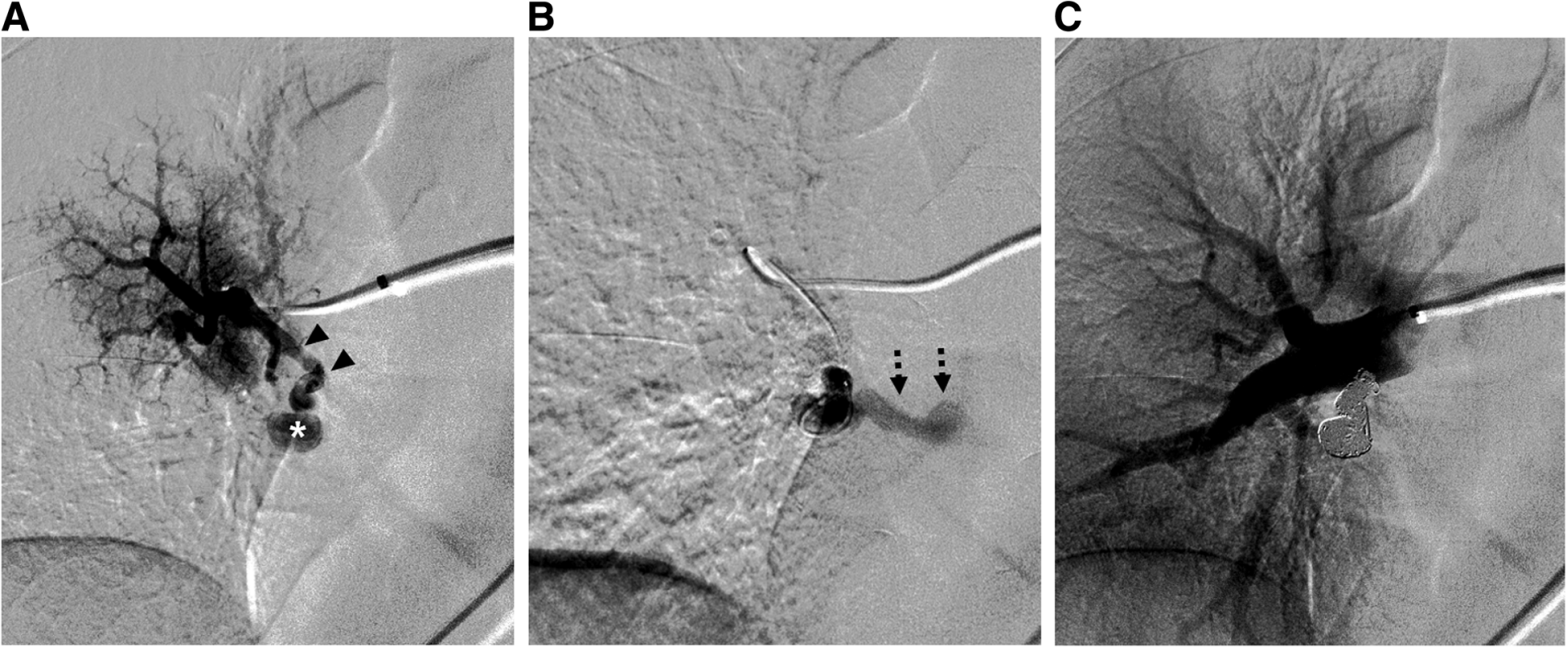
**A** Selective right lower lobe superior segmental pulmonary arteriography shows an acute-angled and feeding artery (arrowheads) and venous sac (asterisk) without an evidence of residual thromboembolism. **B** The angiography performed on the venous sac of PAVM using a microcatheter shows engorged draining vein (dashed arrows) that directly drained to the left atrium. **C** After the venous sac embolization using multiple coils, completion angiography shows no residual shunt flow of the PAVM

**Fig. 3:**
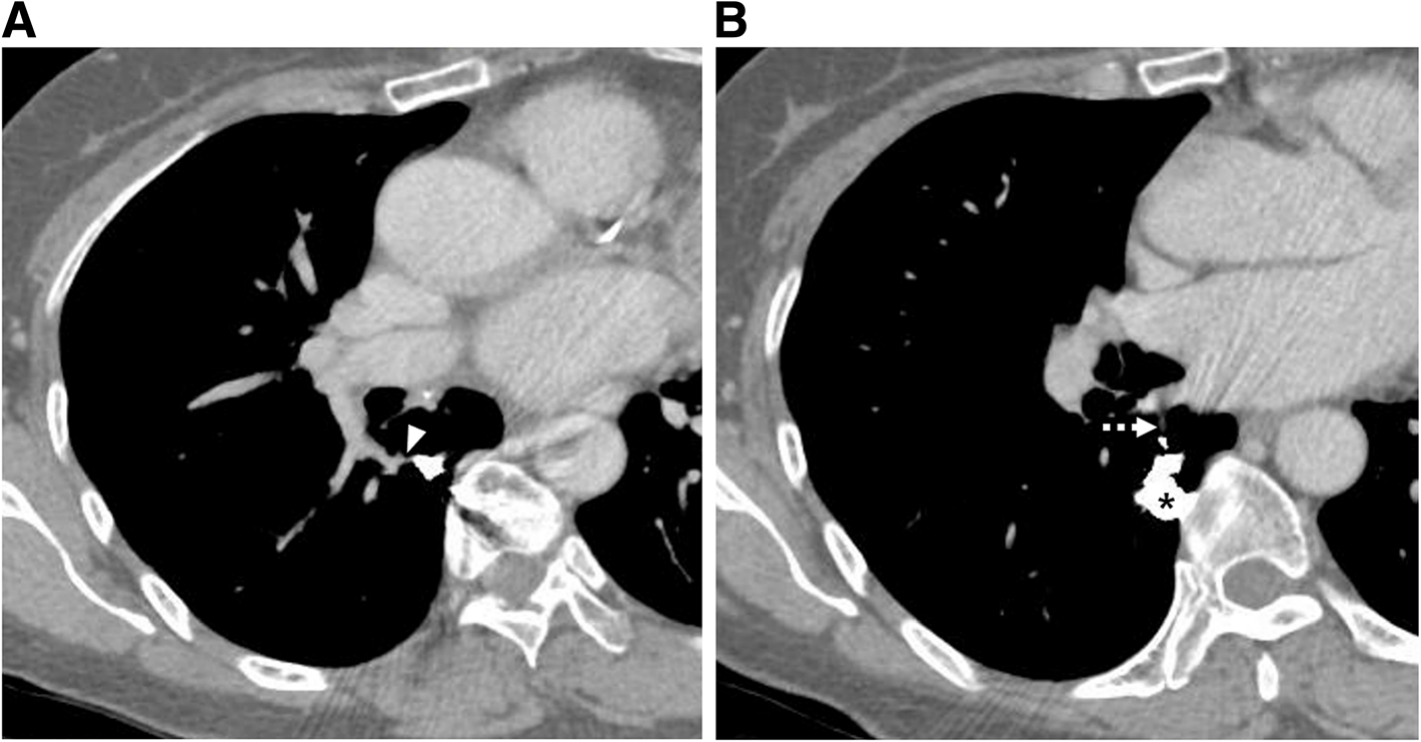
**A, B** After five months of PAVM embolization, the venous sac packed with coil nest (asterisk) and a significant reduction in the size of the feeding artery (arrowhead) and draining vein (dashed arrow) are observed on reconstructed CT images

A 62-year-old female patient was transferred to our hospital with massive PE discovered during hospitalization due to enteritis. She had a history of seven spontaneous abortions and complained of worsening dyspnea for 6 days. Her vital sign was stable, and 97% oxygen saturation was noted on 2 L/min nasal cannula. On chest CT, multifocal PE was observed in the bilateral lobar pulmonary arteries and its segmental branches (Fig. 1A). In addition, a 9 by 6 mm sized solitary simple PAVM with a 3 mm diameter feeding artery was identified in the right lower lobe superior segment (Fig. 1B, C). The patient did not show any other symptoms, signs, or family history that could suggest HHT other than a PAVM. Meanwhile, the acute thromboembolus was extended to the segmental artery where the feeding artery originated (Fig. 1B). Subsequent duplex ultrasound showed no evidence of deep vein thrombosis in both lower extremities. Using enoxaparin, the patient’s dyspnea gradually improved, but on the fifth day of hospitalization, she complained of a sudden onset of left sided weakness. Brain magnetic resonance imaging revealed an acute infarction in the right lateral thalamus and occlusion of the right posterior cerebral artery P2 segment. In such complicated situation, if PAVM embolization was immediately performed, the possibility of thrombus migration during catheter manipulation was expected to be high. After a multidisciplinary discussion, it was decided to embolize the PAVM after sufficiently resolving the PE with anticoagulants. Anticoagulation for a total of 4 months was performed as follows. While monitoring the INR level, the patient took 5–7 mg of warfarin per day for 15 days; then, on an outpatient basis, this was changed to 30 mg of rivaroxaban per day for 21 days and 20 mg per day for the rest of the period. Then, subsequent follow-up CT showed a complete resolution of PE. There were no new-onset neurologic complications during this period. An endovascular treatment was decided, and the patient was referred to the angio suite. After a right common femoral venous access, a 6-Fr guiding catheter (Flexor Shuttle Guiding Sheath; Cook Medical, Bloomington, Indiana) and 5-Fr catheters (Torcon NB Advantage, MPA and Headhunter type; Cook Medical, Bloomington, Indiana) were used to select the right main pulmonary artery. On angiography, the PAVM with a feeding artery arising from the superior segmental artery of the right lower lobe was observed, and no residual PE was noted (Fig. 2A). Then, a microcatheter (Masters Parkway Soft; Asahi Intecc, Tokyo, Japan) was advanced into the venous sac (Fig. 2B), and 10 mm- to 4 mm-sized 11 detachable coils (Concerto; Medtronic, Minneapolis, Minnesota) were used to embolize the venous sac and feeding artery. Completion angiography showed an occlusion of the shunt flow (Fig. 2C). After 5 months, follow-up CT showed a significant reduction in the draining vein and feeding artery diameter, and no recurrence of PE (Fig. 3A, B). With the suspicion of antiphospholipid syndrome (APS) because of the patient’s history, antibody testing was performed during hospitalization and outpatient follow-up, and she was finally diagnosed with APS, with a strong positivity for anti-β2 glycoprotein 1 IgM.

## Discussion

The acute PE in a patient with PAVM is a predisposing factor for life-threatening complications, such as stroke, and there have been few reports of cases in which they were found at the same time and how they were treated (Graves et al., 2009; Serra et al., 2015; Luis et al., 2021). Although HHT, a disease related to PAVM, has been reported to be associated with an increase in thrombotic risk due to iron deficiency (Shovlin et al., 2014), patients with both PAVM and PE are rare. This is a devastating situation, as PE itself can cause hemodynamic instability, and it is a risk for the paradoxical embolism of thromboemboli.

According to Graves et al. (Graves et al., 2009), the acute on chronic PE in a patient with multiple-infarct dementia due to a large PAVM was successfully treated with concurrent anticoagulation and endovascular embolization. Considering the nature of PE, they judged that the risk of migration through minimal catheter manipulation was not high, and the benefit from blocking the shunt was greater. In a report of Serra et al. (Serra et al., 2015), asymptomatic PE was found in the feeding artery course of residual PAVM during a follow-up consultation after multiple PAVM embolization. The remaining PAVM was treated with delayed embolization after one week of anticoagulation. It seems that a safer method was chosen because the burden and clinical significance of PE in this patient were not large.

However, in the current case, a large amount of PE was extended to the segmental artery where the feeding artery of PAVM originated, making it difficult to determine the treatment sequence. Anticoagulation was started while discussing the timing of endovascular embolization. However, stroke due to paradoxical embolism that occurred a few days after hospitalization could not be prevented. Here, several factors that influenced the occurrence of such paradoxical embolism can be postulated. First, considering the history and CT appearance of the PE, it was judged to be an acute type and unstable. Second, the location of the thromboembolus was extended to the segmental artery feeding the PAVM, so there was a possibility of migration by marginal flow or additional thrombosis. Lastly, paradoxical embolism was reported as more common in high-grade shunts on TTCE (Velthuis et al., 2013), which were more likely to be associated with identifiable PAVMs seen on CT (Velthuis et al., 2014). Grade 2 and 3 shunts had odds ratios of 4.78 and 10.4, respectively, compared to grade 1 shunt that had no increased risk. Although it was not confirmed by TTCE in this case, the presence of high grade shunting was highly suspected. Considering these circumstances, it was determined that inducing thrombus fragmentation and migration during catheter manipulation would be riskier than slowly dissolving the thrombus through anticoagulation.

APS is an autoimmune disorder that promotes a hypercoagulable state and causes vascular thrombosis like venous thrombosis, and its most common pulmonary manifestation is PE (Farmer-Boatwright et al., 2009; Sarinc Ulasli et al., 2021). Although it has been reported that an APS-induced PE rarely appears in a massive and life-threatening form (Sarinc Ulasli et al., 2021), its cumulative frequency due to its recurrent nature was reported to be as high as 14.1% (Cervera et al., 2009). Therefore, if it is accompanied by PAVM as in this case, the risk of fatal stroke due to paradoxical embolism will also increase cumulatively. Therefore, an early diagnosis and appropriate treatment are very important.

## Conclusion

The co-occurrence of PAVM and PE is a rare condition. Therefore, the sequence of management for this situation is not established. From the current evidence, it should be adjusted individually according to the patient’s comorbidities and the anticipated risk of thrombus migration due to the age and location of the thrombus or the grade of the PAVM shunt.

## Data Availability

Not applicable.

## Abbreviations

PAVM: Pulmonary arteriovenous malformation
HHT: Hereditary hemorrhagic telangiectasia
TTCE: Transthoracic contrast echocardiography
CT: Computed tomography
PE: Pulmonary embolism
APS: Antiphospholipid syndrome
